# A dataset of human and AI-generated rubric evaluations for formative programming assessment

**DOI:** 10.1038/s41597-026-07397-8

**Published:** 2026-05-25

**Authors:** Pedro C. Mendonça, Filipe Quintal, Karolina Baras, Fábio Mendonça

**Affiliations:** 1 Faculty of Exact Sciences and Engineering, University of Madeira, 9000-082 Funchal, Portugal; 2 Interactive Technologies Institute (ITI/LARSyS) and ARDITI, 9020-105 Funchal, Portugal

## Abstract

Formative assessment in education is often constrained by the time required for rubric design and manual evaluation, limiting the frequency of feedback provided to students. The HARMOGEN-R dataset was created to support research on automated rubric generation and evaluation using artificial intelligence under teacher supervision. It contains evaluations from a Data Structures and Algorithms programming course in which 770 student responses were assessed with five rubrics: one human-created and four generated by large language models (LLMs) using reasoning-enhanced structured and free-form approaches. Each response was evaluated by both human instructors and LLMs, with complete reasoning traces and generated feedback reports recorded to document assessment logic and communication with learners. The dataset also incorporates a variance-based quality control system that flags evaluations exceeding predefined consistency thresholds. This parallel evaluation design enables comparisons of rubric performance, evaluator agreement, and assessment reliability. The dataset is intended for reuse in studies of rubric generation, feedback quality, and automated formative evaluation.

## Background & Summary

Educational assessment in computer programming courses faces global challenges with expanding enrollment numbers^1^. Manual evaluation of code-based assignments requires instructors to assess functional correctness, code quality, algorithmic efficiency, and compliance with programming standards^2^. These evaluation requirements and the need for timely feedback in formative assessment contexts create workload demands that force instructors to choose between assessment frequency and feedback quality^3–5^. This burden is particularly important in foundational programming courses where formative assessments are important for student learning but require substantial time investment^6^.

Assessment reliability also raises concerns. It has been documented that there is considerable variability between human evaluators^2,7,8^, which may influence assessment validity when comparable work receives different grades depending on the evaluator^9^. Even when institutions employ detailed rubrics, interpretation differences and evaluator fatigue contribute to scoring variation^10,11^. Creating these rubrics demands domain expertise, pedagogical knowledge, and iterative refinement based on student performance patterns^12,13^. Reviews and meta-analyses confirm that rubric development requires extensive design time, calibration, and revision cycles, limiting the frequency and diversity of assessments that institutions can implement^14–16^.

Large Language Models (LLMs) have been increasingly investigated as tools for automated educational assessment^1,2,17^. Prior studies reported high correlations between LLM and human evaluations for structured programming tasks, with performance varying across open-ended responses^2,17^. Advances such as chain-of-thought prompting and few-shot learning have improved LLM assessment accuracy, sometimes reaching outcomes comparable to human evaluators^18,19^. However, these approaches remain dependent on the quality and structure of rubrics guiding the assessment process^20,21^.

It is important to note that existing datasets in automated educational assessment primarily target specific domains or task formats. Specifically, the ROARs (Rising and Oxford African Reading Comprehension Short Answer) dataset addresses reading comprehension with human annotations^17^. Furthermore, the RiceChem (Rice University chemistry) dataset provides long-answer chemistry questions for factual evaluation^22^, while the ASAP (Automated Student Assessment Prize) dataset includes essays scored by human raters for automated essay scoring systems^23^. These resources do not address rubric generation or systematic comparison across multiple evaluator types and rubric sources. New research on rubrics generated with Artificial Intelligence (AI) also demonstrates that LLMs can produce evaluation frameworks when provided with learning objectives and example responses^24,25^. Nevertheless, rubric quality influences assessment validity across evaluator categories, making systematic comparison between generation approaches necessary^7^.

**Fig. 1:**
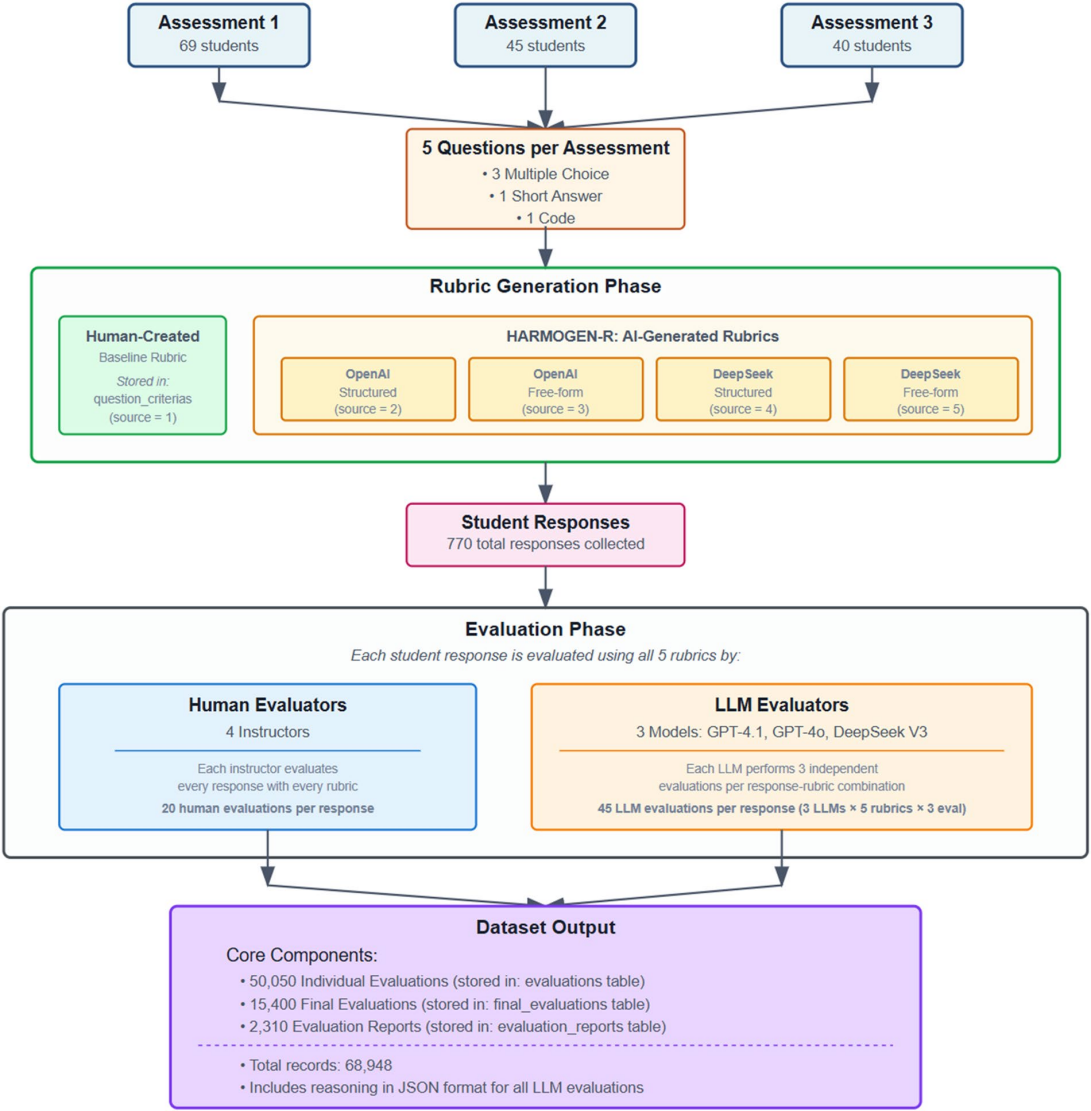
Experimental design and dataset generation workflow. The figure summarizes the multi-phase process, including assessment collection (n = 69, 45, and 40 students for assessments 1, 2, and 3, respectively), rubric generation through HARMOGEN-R (five rubrics, with one human-created and four AI-generated), parallel evaluation by human and LLM evaluators, and the resulting dataset structure containing 68,948 records across 15 interconnected tables.

The HARMOGEN-R dataset was developed to address this gap. It captures evaluations produced through the Hybrid Assessment Rubric Model Generation with Reasoning (HARMOGEN-R) framework, which combines automated rubric generation with multi-dimensional evaluation comparison. This dataset is, to our knowledge, the first to (1) systematically compare AI-generated rubrics with human-created baselines, (2) evaluates the same student responses using multiple rubric sources across both human and LLM evaluators, and (3) records evaluation reasoning to enable analysis of assessment logic beyond numerical scores. An overview of the HARMOGEN-R evaluation workflow and rubric generation process is presented in Fig. 1.

The dataset originates from an evaluation study in a Portuguese university’s Data Structures and Algorithms course during the 2024–2025 academic year. A total of 69 students participated across three formative assessments, producing 770 graded responses, with 345 in the first assignment (69 responses × 5 questions), 225 in the second (45 responses × 5 questions), and 200 in the third (40 responses × 5 questions). The declining participation reflects attrition patterns frequently reported in programming courses^26^. While the dataset reflects 69 student participants, its design captures 770 responses and more than 50,000 individual evaluations with detailed reasoning and feedback, providing a rich foundation for large-scale analyses at the evaluation level. Each assignment maintained a consistent design with five questions, with three multiple-choice (conceptual understanding), one short-answer (conceptual explanation), and one code-based (implementation).

Each student response was evaluated with five rubrics: one human-created baseline and four AI-generated variants. These AI-generated rubrics were produced with two LLMs (OpenAI and DeepSeek), each generating both a structured version (using predefined criteria) and a free-form version (where the model defined criteria, weightings, and scoring rules). This allowed for a comparison between generation approaches.

The inclusion of multiple rubric sources reflects the established finding that rubric design influences evaluation outcomes. Prior research has shown that even when detailed rubrics are employed, interpretation differences and evaluator-specific factors contribute to scoring variation^2,8,27^, and that rubric quality affects assessment validity across evaluator categories^10^. Furthermore, recent work has demonstrated that LLM scoring behavior varies with the rubric-related information provided, indicating that the rubric source and the evaluator interact in determining assessment outcomes^20^. The application of five rubrics to the same set of student responses allows the dataset to address three complementary lines of investigation. First, the agreement between human and AI-generated rubric-based evaluations, examining whether AI-generated rubrics produce scoring outcomes comparable to human-created baselines. Second, the sensitivity of grading results to different rubric designs, including the distinction between structured rubrics with predefined criteria and free-form rubrics where the model defines the evaluation framework. Third, the potential of LLMs to generate effective assessment rubrics, comparing the outputs of open-source and proprietary models across both generation approaches. The inclusion of evaluation reasoning traces in the *criteria_evaluations_by_llm* field further supports analyses of how different rubric structures influence the assessment logic applied by automated evaluators.

Evaluation was performed by human and automated assessors. Human evaluation involved four programming instructors independently applying all rubrics to each response, yielding 20 human evaluations per submission. Automated evaluation employed three LLMs (GPT-4.1, GPT-4o, and DeepSeek V3), each completing three independent assessments per response–rubric combination, producing 45 automated evaluations per response. An automated quality-control mechanism flagged evaluations for review when discrepancies appeared among the three independent LLM assessments, providing reliability indicators.

The dataset is stored in a relational database with 15 interconnected tables comprising 68,948 records. It includes numerical scores, categorical variables identifying question type (multiple-choice, text, code), and timestamps for submissions and evaluations. Structured text fields contain evaluation reasoning in JavaScript Object Notation (JSON) format. Relational mappings through foreign keys link students, responses, evaluations, and rubrics. The dataset contains 50,050 individual evaluations, 15,400 final consensus evaluations, and 2,310 evaluation reports. Criterion-level breakdowns show how rubric components contribute to overall assessment, while consensus evaluations synthesize multiple evaluator inputs through algorithmic aggregation.

This structure allows the investigation of fundamental questions about AI-generated rubrics, namely, the equivalence with human-created rubrics, variation across evaluator types, and characteristics that influence evaluation consistency. Including reasoning data supports analyses of how evaluators interpret and apply criteria^28,29^. The Structured Query Language (SQL) structure and JSON formatting allow researchers to extract targeted subsets for tasks such as training new automated assessment models using the evaluation examples, evaluating rubric effectiveness through criterion-level data, or investigating evaluator bias patterns. Including proprietary (GPT-4.1, GPT-4o) and open-source (DeepSeek V3) models further supports cost–benefit and data sovereignty analyses for institutions exploring automated assessment^30–32^.

Making this dataset publicly available enables reproducible research in AI-enhanced educational assessment. Furthermore, documentation of data collection, rubric generation, and evaluation protocols ensures transparency and reusability.

## Methods

### Ethics statement

The study received ethical approval from the relevant Ethics Commission (N° 169 A/CEUMA/2025). Participants were undergraduate students enrolled in the Data Structures and Algorithms curricular unit at the University of Madeira. All participants were aged 18 years or older. As no minors were involved in the study, parental consent was not required. Recruitment was conducted by the lead researcher during regular class sessions. Students were informed about the study’s purpose, procedures, and the voluntary nature of participation. A detailed explanation was provided, and information sheets and consent forms were distributed to all eligible students. Those who agreed to participate signed the consent form, which covered involvement in the study, including the collection of responses and their evaluation by both human and automated assessors. The public release of the anonymized dataset was subsequently approved by the Ethics Committee through a formal addendum to the original approval (Addendum to Parecer N° 169 A/CEUMA/2025).

To protect participant information, the platform collects only institutional email addresses for authentication purposes, which are composed of a number that is only identifiable inside the university database (the emails are in the format “student_number@student.uma.pt”). Then, all student responses and evaluation records are linked to internal numeric identifiers rather than personal data, ensuring that the released dataset contains no personally identifiable information. Data are stored in a secure server-hosted database with access restricted to the research team. Users must comply with the license terms (CC BY 4.0) and cite the dataset appropriately in any publications resulting from its use.

### Study design

The dataset was generated through a longitudinal educational assessment study employing a within-subjects experimental design, in which five rubrics were applied to evaluate the student responses, collected across three formative assessments in a Data Structures and Algorithms curricular unit. Each response was assessed by four human instructors and three LLMs, with each LLM performing three independent evaluations per response–rubric combination, enabling systematic comparison across rubric source, generation approach, and evaluator type, as shown in Fig. 1.

### HARMOGEN-R Framework

**Fig. 2:**
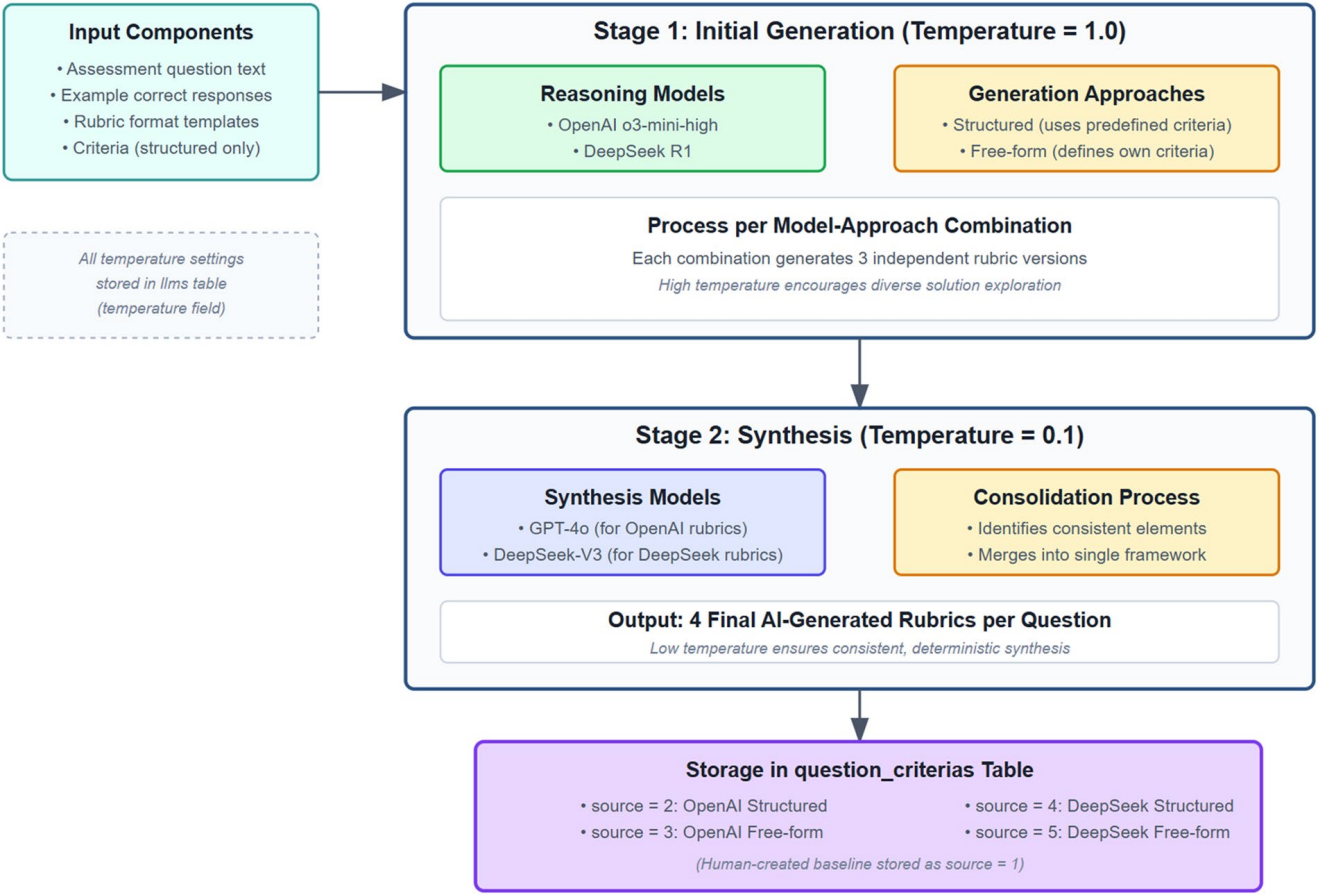
The HARMOGEN-R framework for AI-generated rubric creation. Stage 1 used reasoning models (OpenAI o3-mini-high, DeepSeek R1) to generate structured and free-form rubrics. Stage 2 synthesized three independent versions per model–approach combination using consolidation models (GPT-4o, DeepSeek V3). Final rubrics were stored in the question_criterias table with source identifiers.

The HARMOGEN-R framework implements a two-stage process for generating assessment rubrics using LLMs, as presented in Fig. 2. The framework produces four AI-generated rubrics, stored alongside a human-created baseline. In Stage 1, reasoning-enhanced LLMs (OpenAI o3-mini-high and DeepSeek R1) generated rubrics through two strategies. In the structured version, models received predefined evaluation criteria with instructor-assigned score weights and produced detailed scoring rules for each criterion. In the free-form version, models defined their own evaluation criteria, assigned weightings according to concept importance, and generated corresponding scoring rules. Each model produced three independent rubric versions per assessment question, using a temperature setting of 1.0 to promote variation in criterion formulation and scoring structure across the generated candidates.

Stage 2 consolidated these initial versions using standard LLMs (OpenAI GPT-4o for OpenAI-generated rubrics and DeepSeek V3 for DeepSeek-generated rubrics) operating at a temperature of 0.1 to reduce output variability and ensure consistent synthesis. The temperature parameter controls the degree of randomness in LLM outputs. Higher values encourage diverse responses, and lower values produce more deterministic results. This step identified consistent elements across versions and merged them into a single rubric framework.

All generated rubrics were stored in the *question_criterias* table with source identifiers: source = 1 (human-created), source = 2 (OpenAI structured), source = 3 (OpenAI free-form), source = 4 (DeepSeek structured), and source = 5 (DeepSeek free-form). Each rubric entry includes the criterion title, description, scoring rules (*score_criteria* field), point values, and timestamps. The scoring system employs variable scales defined at two levels: assignments specify maximum total scores in the *assignments* table (*total_score* field, set to 200 points in this study), while individual questions define their allocated points in the *questions* table (*score* field). This hierarchical scoring structure accommodates different point distributions across question types, with scores subsequently normalized to a 0–100 scale for comparative analysis as described in the Data Records section. Temperature settings used during generation are recorded in the *llms* table (*temperature* field).

### Data collection procedures

Student responses were collected through a web-based platform with institutional authentication, capturing submissions in the student_responses table with timestamps and unique constraints preventing duplicates. The platform preserved text formatting through UTF-8 encoding and maintained code structure, including whitespace and indentation. The collection occurred over three assessment periods during the 2024−2025 academic semester, and a more detailed description of the platform design and workflow is provided in Mendonça *et al*.^2^.

Human evaluations followed a blind review protocol with four instructors accessing responses in randomized orders unique to each evaluator-rubric combination. Each instructor evaluated all responses using all five rubrics sequentially. Evaluations were stored in the *evaluations* table with evaluator_type = ‘teacher’ and linked through *evaluator_id* foreign keys. Automated evaluations employed structured prompts containing the question, rubric criteria from the *question_criterias* table, exemplar responses from the *question_response_examples* table, and the target response. Text questions incorporated Retrieval-Augmented Generation (RAG) using vectorized course materials stored in the *rag* table, while code questions underwent direct evaluation. Each LLM performed three independent evaluations with results stored as JSON in the *criteria_evaluations_by_llm* field.

Quality control flagged cases where the standard deviation exceeded 10 points across the three LLM iterations, updating the *need_teacher_review* field in the *final_evaluations* table. This table aggregated scores using mean calculation for human and LLM evaluators (stored in the *final_score* field). For LLM evaluations, the system additionally stored synthesized feedback from the three iterations in the *final_feedback* field. Student–assignment-level evaluation reports were recorded in the *evaluation_reports* table, containing weighted averages and criterion-level performance breakdowns. The process produced 50,050 individual evaluation records, comprising 15,400 human evaluations (770 responses × 4 instructors × 5 rubrics) and 34,650 LLM evaluations (770 responses × 3 models × 5 rubrics × 3 iterations). The final_evaluations table contains 15,400 aggregated records: 3,850 for human evaluators (770 responses × 5 rubrics, averaging across the four instructors) and 11,550 for LLM evaluators (770 responses × 5 rubrics × 3 models, averaging across the three iterations per model). The evaluation_reports table stores 2,310 assignment-level summaries (770 responses × 3 LLM models). Together with the supporting metadata tables described in Table 1, the dataset comprises 68,948 records across 15 interconnected tables (Fig. 1).

**Table 1:** Overview of the 15 tables in the HARMOGEN-R dataset, including purpose, record counts, and key relationships.

Table Name	Description	Foreign key references	Records
assignments	Metadata for the three formative assessments, defining submission deadlines and maximum scores (total_score).	uc_school_year_id** → **uc_school_year.id	3
questions	Assessment items (9 multiple-choice, 3 short-answer, 3 code-based) with scoring weights and type classification.	assignment_id → assignments.id	15
question_criterias	Rubrics: one human baseline and four AI-generated (structured or free-form) per question, with scoring rules and weights.	question_id → questions.id	168
question_response_examples	Reference responses with associated scores used in LLM prompts for few-shot learning.	question_id → questions.id	22
student_responses	Student submissions preserving text formatting and code indentation.	question_id → questions.id	770
evaluations	All evaluation records: 15,400 human and 34,650 LLM assessments with scores, feedback, and criterion-level JSON.	student_response_id → student_responses.id; evaluator_id → llms.id; rag_id → rag.id	50,050
final_evaluations	Aggregated scores for human and LLM evaluators, mean-based with feedback synthesis and variance flag (need_teacher_review).	response_id → student_responses.id; evaluator_id → llms.id;	15,400
evaluation_reports	Assignment-level summaries with overall scores and four feedback components (balance, strengths, weaknesses, suggestions).	assignment_id → assignments.id; evaluator_id → llms.id;	2,310
llms	Configuration of models used in rubric generation and evaluation (model name, API, temperature).	—	14
rag	RAG settings linking assessments to vector databases with course materials.	assignment_id → assignments.id	3
ucs	Course metadata (unit name, year, semester, program).	—	1
uc_school_year	Junction linking curricular units and academic years.	uc_id → uc.id; school_year_id → school_years.id;	1
school_years	Academic year definitions.	—	1
uc_students	Links students to a specific course unit and school year (student enrolments).	school_year_id → school_years.id	185
uc_teachers	Links teachers to a specific course unit and school year (teaching allocations).	school_year_id → school_years.id	5

Each table uses an auto-incremented integer *id* as the primary key. Foreign-key references indicate links within the relational schema; *uc_students* and *uc_teachers* associate learners and instructors with specific course units and years.

**Fig. 3:**
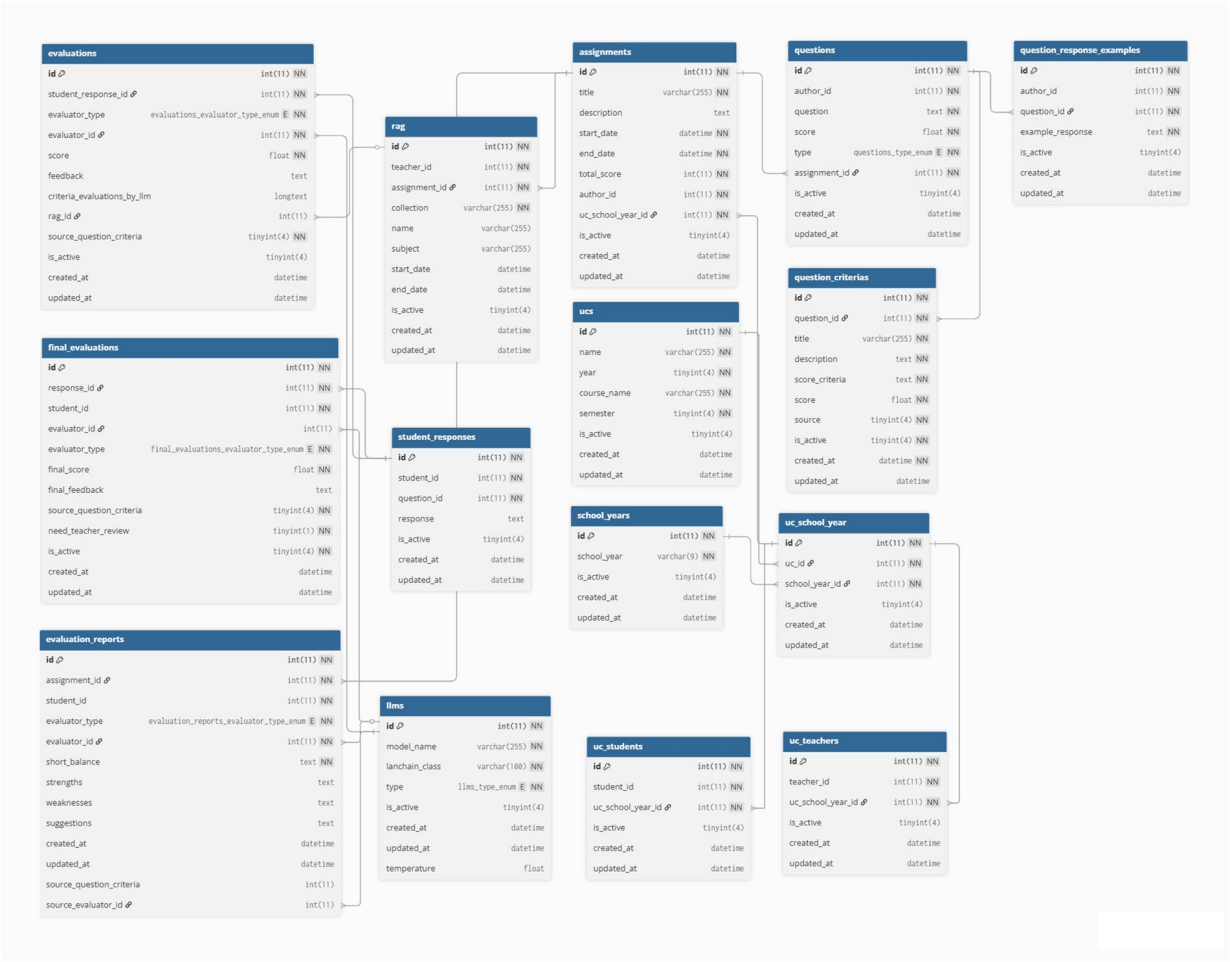
Entity–relationship diagram of the HARMOGEN-R dataset, showing the relational database structure, comprising 15 interconnected tables. It also shows how tables storing responses, rubrics, and evaluations are linked through foreign keys, supporting data integrity and enabling systematic comparison across rubric sources, evaluator types, and assessment periods.

Figure 3 illustrates the complete database architecture through an entity-relationship diagram, showing how the 15 interconnected tables support the data collection and evaluation workflow, while Table 1 provides descriptions of each table’s purpose and contents, documenting the distribution of 68,948 records across the dataset structure.

Table 2 documents all dataset fields whose values are coded or whose semantics may not be immediately apparent from the field name alone. Fields with self-explanatory names are not listed. Complete field definitions and data types are available in the database schema file (*harmogen_dataset_schema.sql*) provided in the dataset repository^33^.

**Table 2:** Description of dataset fields with coded or non-obvious values.

Table(s)	Field	Description
question_criterias	source	Rubric source identifier: 1 = human-created baseline; 2 = OpenAI structured; 3 = OpenAI free-form; 4 = DeepSeek structured; 5 = DeepSeek free-form.
evaluations, final_evaluations, evaluation_reports	source_question_criteria	Same encoding as question_criterias.source; identifies which rubric was applied in the evaluation.
evaluations, final_evaluations, evaluation_reports	evaluator_type	Evaluator category: ‘teacher’ for human instructors; ‘llm’ for language model evaluators.
evaluations, final_evaluations, evaluation_reports	evaluator_id	References uc_teachers.teacher_id when evaluator_type = ‘teacher’; references llms.id when evaluator_type = ‘llm’.
evaluations	criteria_evaluations_by_llm	JSON object containing criterion-level evaluation data for LLM assessments, including individual scores, textual justifications, and confidence metrics for each rubric criterion. NULL for human evaluations.
final_evaluations	need_teacher_review	Quality-control flag: 0 = no review required; 1 = flagged for teacher review when the standard deviation across the three LLM iterations exceeded 10 points.
questions	type	Question format: ‘text’ for short-answer and multiple-choice questions; ‘code’ for programming questions; ‘longtext’ for extended-response questions.
llms	type	Model licensing category: ‘open-source’ or ‘premium’.
llms	lanchain_class	Name of the LangChain Python class used to interface with the model API (e.g., ChatOpenAI, ChatDeepSeek).

Fields with self-explanatory names (e.g., id, title, score, created_at, feedback) are not listed, as their definitions can be consulted in the database schema file available in the dataset repository.

### Dataset structure and access

The HARMOGEN-R dataset is distributed in three formats to support different research environments. The primary format is a relational SQL database, which preserves the full structure of 15 interconnected tables with foreign key constraints, ensuring referential integrity. To enable use without requiring a database system, the dataset is also provided as individual Comma-Separated Values (CSV) and JSON files (one per table), exported with UTF-8 encoding. In the CSV files, the *criteria_evaluations_by_llm* field is stored as a JSON string in the corresponding column. In the JSON files, it is represented as a native JSON object with fully parsed nested structures. The dataset contains extensive text strings, including multi-paragraph student responses, code submissions with special characters and indentation patterns, and detailed evaluation feedback, all of which are fully preserved across the three distribution formats.

Language considerations also required explicit documentation for dataset users. The database schema, including table names, field names, and structural elements, is implemented in English to ensure international accessibility and compatibility with standard database tools. However, all content data remains in Portuguese as collected initially. This includes student responses, evaluation feedback from human and LLM evaluators, rubric descriptions, scoring criteria, and exemplar responses for few-shot learning. This bilingual structure reflects the study’s origin at a Portuguese university while facilitating international research use.

Access to the dataset requires a MySQL-compatible database system or equivalent SQL engine capable of importing the provided*.sql* dump file. The JSON fields require MySQL 5.7 or higher for native JSON support. In earlier versions, the data remains accessible as text fields but requires manual parsing.

## Data Records

The dataset is publicly available at Zenodo^33^ under a CC BY 4.0 license. Full documentation and import instructions are provided in the accompanying *README* file. To support different research environments, the dataset is distributed as a single archive containing multiple format options:

1. *sql/* — SQL dump files, including *harmogenr_dataset.sql* (single file with all 68,948 records), *harmogenr_tables.zip* (individual SQL files for modular import), and *harmogenr_dataset_schema.sql* (schema only);
2. *csv/tables/ —* individual CSV files for each of the 15 tables, with UTF-8 encoding, enabling direct use in statistical tools without requiring a database system*;*
3. *json/ —* individual JSON files for each of the 15 tables, with the *criteria_evaluations_by_llm* field represented as native JSON objects;
4. *csv/ —* pre-processed analytical CSV extracts with normalized scores, plus *evaluations_criteria_expanded.csv* providing a flattened view of criterion-level evaluation data.

The package also includes *harmogen_dataset_schema.sql*, containing the database structure without data, enabling users to inspect the schema before importing the full dataset. The README file provides import instructions and basic usage examples. The SQL dataset can be examined with any SQL client or database browser, while the CSV and JSON files can be loaded directly into programming environments such as Python or R for efficient data handling.

Extracted CSV files are available for users who require tabular data suitable for statistical analysis. These files correspond to analytical views exported from the relational database, as the complete dataset, which contains JSON fields and relational links, cannot be directly represented as flat tables. The file *all_evaluations_wide_format_all_assignments_normalized.csv* (3,850 rows) contains response–rubric combinations with normalized scores on a 0 to 100 scale across all evaluators. Normalization was applied per question using the formula: Normalized Score = (Raw Score / Q_max) × 100, where Q_max denotes the maximum score defined for each question in the questions table (score field). This linear rescaling preserves the original score distributions while enabling direct comparison across questions with different point allocations. Two additional subsets, *all_evaluations_wide_format_assignment_7_normalized.csv* (1,725 rows) and *rubric_comparison_final_evaluations_assignment_7_normalized.csv* (6,900 rows), provide assignment-level data for independent analyses. These CSV files are derived subsets designed for statistical applications and do not include the complete JSON reasoning data, full rubric content, or student response texts contained in the SQL database. To illustrate the content of the dataset, Tables 3, 4, and 5 present representative examples for each of the three question types included in the assessments. Each table shows the question text, a sample anonymized student response, and the corresponding evaluation criteria from the human-created baseline rubric. All content has been translated from the original Portuguese. Table 3 illustrates a multiple-choice question, Table 4 a short-answer text question, and Table 5 a code-based question.

**Table 3:** Representative example of a multiple-choice question (20 points), including the question text, a sample student response, and the human-created baseline rubric criteria.

Question (translated)	Student Response (example)	Rubric Criteria (Human baseline)
Assume that we declare the following array: double valores[5] = {3.5, 7.2, 1.8, 9.4, 2.1}; If the first element is stored at memory address 1000, what will be the address of the third element? Indicate the corresponding option: a) 1008 b) 1016 c) 1024 d) 1032	b) 1016	- If option b) or the value 1016 is indicated: 20 points. - If another option or a value different from the correct one is indicated: 0 points.

Content translated from Portuguese.

**Table 4:** Representative example of a short-answer text question (50 points), including the question text, a sample student response, and the human-created baseline rubric criteria.

Question (translated)	Student Response (example)	Rubric Criteria (Human baseline)
Once declared, is it possible to change the size of an array? Justify your answer and explain what happens if we try to store values outside its bounds.	It is not possible to change the size of an array after it has been declared, since it is a data structure with a fixed size. If we attempt to store a value outside its bounds, the result will be unpredictable, such as program crashes or data corruption.	**Changing the size of the array** - If it is correctly stated that this is not possible: 15 points. If it is stated that it is possible: 0 points. **Justification regarding memory allocation** - Complete and correct description: 25 points. - Partial description (between 5 and 15 points): if the explanation is incomplete or does not cover all relevant aspects. - No answer or significantly incorrect answer: 0 points. **Explanation of storing values outside the bounds of the array** - Complete and correct description: 10 points. - If the occurrence of an overflow is mentioned, the full 10 points should be awarded. - Partial description (between 3 and 6 points): if the explanation is only partially correct. - No answer or significantly incorrect answer: 0 points.

Content translated from Portuguese.

**Table 5:** Representative example of a code-based question (90 points), including the question text, a sample student response, and the human-created baseline rubric criteria.

Question (translated)	Student Response (example)	Rubric Criteria (Human baseline)
a) Implement a C+ + program that: a) Defines two structures: - Address, containing the following attributes: street, postcode, and locality; - Person, containing the following attributes: id, name, date of birth, and an address (of type Address). b) Implements the function inserirPessoa, which must create and return a structure of type Person, filling in all its attributes inside the function itself with values entered by the user. Ensure that the function correctly returns the completed structure. c) In main: - Ask the user how many people they wish to add; - Initialise a dynamic array of people; - Fill the array by calling the function inserirPessoa for each person; - After filling the array, print the total number of registered people; - Make sure to free the dynamically allocated memory to avoid memory leaks. d) Include the necessary libraries for the program to work correctly.	struct Morada{ char rua;^50^ int codigo_postal; char localidade;^50^ }; struct Pessoa{ int id; char nome;^50^ int data nascimento; morada = Morada; }; Pessoa inserirPessoa() { Pessoa = p; cout «"Inisira o seu nome"; cin » (nome); cout «"Qual a sua data de nascimento"; cin » (data_nascimento); cout «"Digite a rua onde mora"; cin » (morada); cout «"Digite o seu código postal"; cin » (codigo_postal); cout «"Qual a sua localidade"; cin » (localidade); return p; } int main() { int pessoas; cout «"Quantas pessoas deseja adicionar? "; cin » pessoas; vetor < Pessoa > pessoas; for (int i = 0; i < pessoas; i + + ) { cout «"Inserindo dados para a pessoa \n" «i + 1 «endl; pessoas(inserirPessoa(i + 1)); } cout «"Número total de pessoas registadas: \n" «endl; return 0;}	**Definition of the Address Structure** - Correctly define the structure and its identifier: 3 points; - Correctly define the 3 attributes (correct type, identifier at the student’s discretion, provided it is descriptive and reasonable): 9 points; - If the student defines one attribute incorrectly (correct type, identifier at the student’s discretion), 3 points should be deducted. If more than 1 attribute is incorrect: 0 points; **Definition of the Person Structure** - Correctly define the structure and its identifier: 3 points; - Correctly define the 4 attributes (correct type, identifier at the student’s discretion, provided it is descriptive and reasonable): 12 points; - If the student defines one attribute incorrectly, 3 points should be deducted. If more than 1 attribute is incorrect: 0 points; **Implementing the inserirPessoa Function** - Correctly define the return type and the identifier of the inserirPessoa function (with no parameters): 6 points; - Initialise a Person structure inside the function: 4 points; - The user must fill in all the person’s attributes (including the address), for a total of 6 attributes: 18 points. Deduct 3 points for each attribute not filled in. If the student fills in fewer than 3 attributes correctly, the answer receives 0 points; - Correctly return the completed Person structure: 5 points; **Implementation of the Instructions in main (30 points)** - Correctly read the number of people (using cin): 2 points; - Correctly initialise a dynamic array (new Person[n]): 6 points; - Fill the array by calling the inserirPessoa function for each person: use a for or while loop to iterate up to n: 6 points; correctly fill one position of the array with a Person returned by inserirPessoa: 6 points; - Correctly print the total number of registered people: 4 points; - Free the dynamically allocated memory (delete[]): 6 points; **General rules:** - Penalty for unnecessary or incorrect elements: −5% to −10% for unnecessary elements that do not affect functionality; −10% to −20% for errors that alter the expected behaviour; - Award 0 points if any of the following occurs: No answer.

Content translated from Portuguese.

To illustrate the structure of the criterion-level evaluation data stored in the *criteria_evaluations_by_llm* JSON field, Table 6 presents an example record corresponding to the code-based question shown in Table 5. This record demonstrates how each LLM evaluation is decomposed into individual criteria with associated scores and textual justifications, enabling fine-grained analysis of assessment reasoning beyond aggregate scores.

**Table 6:** Example of a criterion-level LLM evaluation record extracted from the *criteria_evaluations_by_llm* JSON field in the *evaluations* table.

Criterion	Score	Justification
Definition of the Address Structure (12 points)	9.0	The Address structure is defined with the correct identifier (3 points). Of the three attributes, ‘rua’ and ‘localidade’ are correct (char[] type acceptable), but ‘codigo_postal’ is declared as int when it should be a string to accept postal codes with letters (e.g., 1000-001). Thus, 2 correct attributes (6 points), 1 incorrect (−3 points). Total: 3 + 6 = 9 points.
Definition of the Person Structure (15 points)	0.0	The Person structure has several errors: ‘data nascimento’ is separated and is not a valid identifier, the correct type is missing (should be string or char[]), and the address declaration is incorrect (‘morada = Morada;’). Since more than one attribute is incorrect, 0 points are assigned per the criterion.
Implementation of the inserirPessoa Function (33 points)	0.0	The function has multiple critical errors: the Person variable declaration is wrong (‘Pessoa = p;’), attribute access does not use the structure (e.g., ‘cin » (nome);’ instead of ‘cin » p.nome;’), and the address attributes are not correctly populated. Fewer than 3 attributes are correctly filled, resulting in 0 points.
Implementation of the Instructions in main (30 points)	2.0	The program correctly reads the number of people using cin (2 points). However, the dynamic array initialisation is wrong (‘vetor < Pessoa > pessoas;’), new is not used, and there is no memory deallocation. The for loop is present but the call to inserirPessoa is incorrect and does not fill the array properly. The total number of people is not printed correctly. Only the reading of the number of people is correct.

This entry corresponds to the code-based question presented in Table 5, evaluated using the human-created baseline rubric (source = 1) by GPT-4.1 (evaluator_id = 1). The overall score for this evaluation was 11.0 out of 90 points. Content translated from Portuguese.

**Fig. 4:**
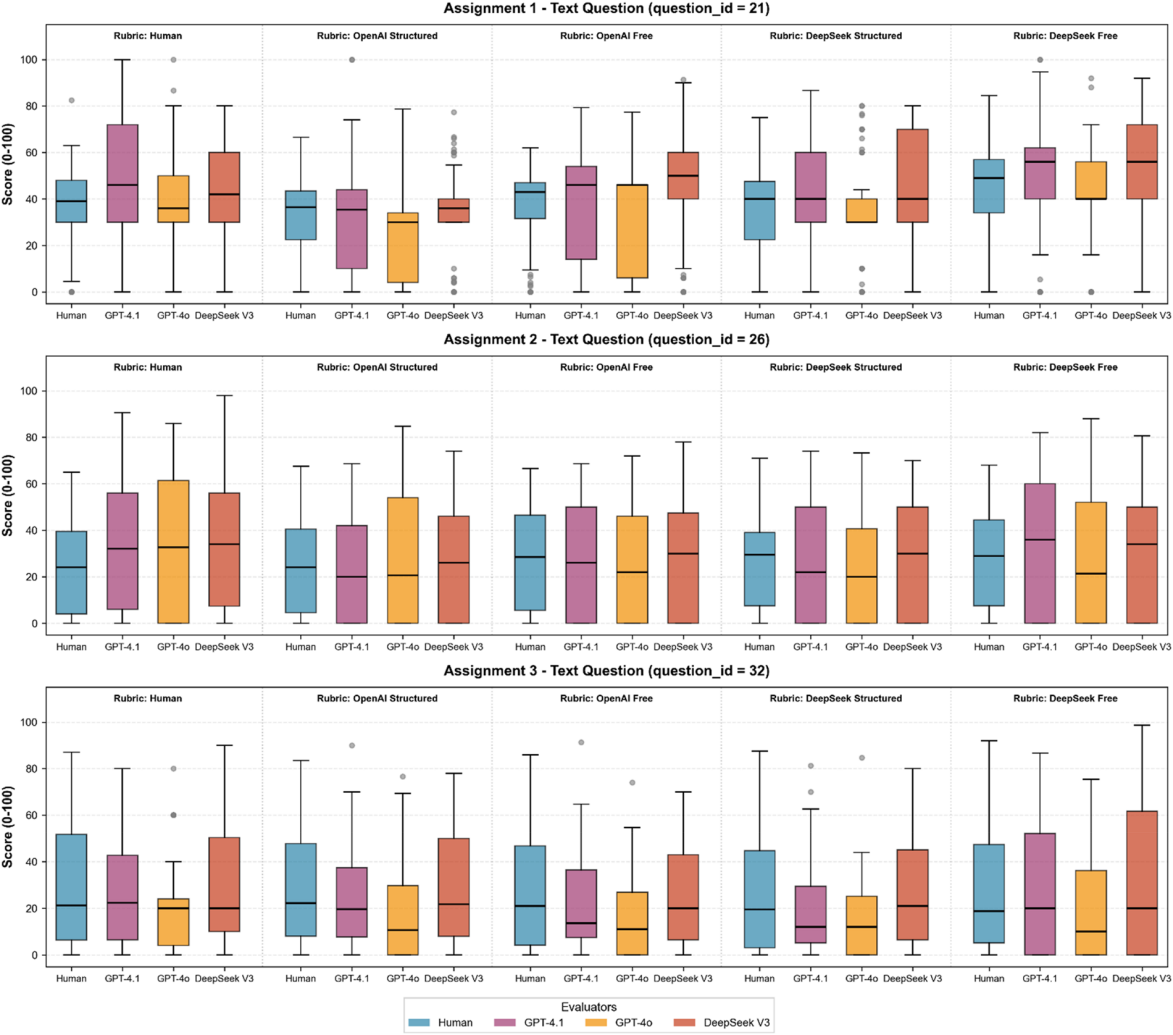
Distribution of evaluation scores across rubric sources and evaluator types for text questions. The boxplots show score distributions (0–100 scale) for four evaluators (Human, GPT-4.1, GPT-4o, and DeepSeek V3) across five rubric variants (Human-created, OpenAI Structured, OpenAI Free, DeepSeek Structured, and DeepSeek Free) for the three text questions (one per assignment).

Regarding the distribution of evaluation outcomes across the dataset, Fig. 4 presents the normalized scores from the CSV extracts for text-based questions across the three assignments.

## Technical Validation

Data integrity was evaluated through several verification procedures. Referential integrity was confirmed by checking foreign key constraints across all fifteen tables, ensuring that no records lacked valid references. Dataset completeness was verified by confirming that all 770 student responses received the expected number of evaluations: twenty human evaluations (four instructors × five rubrics) and forty-five LLM evaluations (three models × five rubrics × three iterations) per response, yielding a total of 50,050 individual evaluation records.

Quality-control procedures identified evaluations requiring human review through activation of the *need_teacher_review* flag in the *final_evaluations* table. This flag was present in 10.89% of cases, indicating that approximately 89% of assessments did not require additional verification.

Analysis of the normalized wide-format extracts indicated high consistency between teacher and LLM evaluations. The Spearman rank correlation between teacher and LLM scores was *r* = 0.991. The mean signed score difference (LLM − teacher) was −2.02%, and more than 90% of paired evaluations were within a ±15% tolerance band. These metrics demonstrate internal consistency across evaluator types, while small variations may occur with alternative extraction or aggregation parameters.

The dataset is balanced across experimental conditions, with 770 evaluations (20%) per rubric source and complete data across all tables. The integrity of the JSON field *criteria_evaluations_by_llm* was verified by parsing all 34,650 LLM evaluation records, confirming the presence of criterion-level scores, textual justifications, and confidence metrics.

## Usage Notes

Researchers using this dataset should note that all content data, including student responses, evaluation feedback, rubric descriptions, scoring criteria, and exemplar responses, are written in Portuguese, reflecting the study’s origin at a Portuguese university. The database schema is in English to facilitate international use. Users who require other languages may apply translation workflows during preprocessing without affecting the structural integrity of the dataset.

JSON fields are natively supported in MySQL version 5.7 or higher. When using earlier versions, these fields are stored as text and must be parsed manually. For users who do not require a database system, the per-table CSV and JSON exports contain the complete information from all 15 tables, including the *criteria_evaluations_by_llm* field with criterion-level scores and textual justifications. The file *evaluations_criteria_expanded.csv* provides a flattened view of this JSON field with one row per criterion, enabling direct statistical analysis without JSON parsing. The accompanying analytical CSV files contain normalized scores on a 0 to 100 scale for cross-question comparisons. These CSV extracts are optimized for statistical analysis, and alternative extraction or aggregation methods may produce slightly different statistical metrics while preserving the general evaluator-agreement patterns.

The dataset includes a quality-control mechanism via the *need_teacher_review* flag, which identifies evaluations with higher variance and can assist studies on assessment reliability or human-oversight requirements.

When working with the JSON field *criteria_evaluations_by_llm*, users should parse nested structures that contain criterion-level scores, textual justifications, and confidence metrics for each of the 34,650 LLM evaluations. The dataset’s relational structure, comprising fifteen interconnected tables with foreign-key constraints, maintains referential integrity but requires familiarity with SQL joins to reconstruct complete evaluation chains, from student responses through rubric application to final scoring.

For optimal use, researchers should consider that each response–rubric combination includes three independent LLM evaluations, enabling analyses of consistency and reliability across models. The inclusion of both structured and free-form AI-generated rubrics allows comparative studies of rubric-generation approaches, while the parallel evaluation structure across five rubric sources and multiple evaluator types supports systematic investigation of rubric effectiveness and evaluator-agreement patterns.

The dataset originates from a single undergraduate computer science course at a Portuguese university, which limits direct generalizability to other educational contexts, disciplinary domains, or student populations. While the number of student participants is smaller than in large-scale machine learning datasets, the within-subjects evaluation design amplifies the analytical value of each response through multiple rubric sources, evaluator types, and criterion-level reasoning traces, as described in the Background & Summary section. The dataset covers three question types within a single programming discipline; applicability to subjects involving interpretive or subjective evaluation remains to be established through future work.

## Data Availability

The HARMOGEN-R dataset is publicly available at Zenodo^33^, under a CC BY 4.0 license. The repository includes the complete SQL database, extracted CSV files for statistical analysis, code examples for rubric generation using the HARMOGEN-R framework, and scripts for data extraction and analysis.

## Code availability

The dataset repository provides all code resources required for data creation, processing, and analysis. The available materials are organized into four categories: (1) database utilities, including *harmogen_dataset_schema.sql*, data-import scripts, and extraction queries for generating analytical views; (2) rubric-generation examples implementing the HARMOGEN-R framework for structured and free-form generation approaches; (3) data-processing scripts for database extraction to CSV (*harmogenr_export_to_datasets_example.py*), score normalization (*harmogenr_normalization.py*), and exploratory data analysis (*harmogenr_exploratory_data_analysis.py*); and (4) a methodological reference script linking to the complete platform implementation described in reference^2^. All scripts contain internal documentation and example commands. The repository *README* file provides detailed instructions for dataset setup, extraction procedures, and analytical workflows, including software dependencies and version requirements.
